# Antibacterial, Immunomodulatory, and Lung Protective Effects of *Boswellia* *dalzielii* Oleoresin Ethanol Extract in Pulmonary Diseases: In Vitro and In Vivo Studies

**DOI:** 10.3390/antibiotics10121444

**Published:** 2021-11-25

**Authors:** Badriyah Alotaibi, Walaa A. Negm, Engy Elekhnawy, Thanaa A. El-Masry, Walaa S. Elseady, Asmaa Saleh, Khalid N. Alotaibi, Suzy A. El-Sherbeni

**Affiliations:** 1 Department of Pharmaceutical Sciences, College of Pharmacy, Princess Nourah Bint Abdulrahman University, Riyadh 84428, Saudi Arabia; bsalotaibi@pnu.edu.eg (B.A.); asali@pnu.edu.eg (A.S.); 2 Department of Pharmacognosy, Faculty of Pharmacy, Tanta University, Tanta 31111, Egypt; suzy.elsherbini@pharm.tanta.edu.eg; 3 Pharmaceutical Microbiology Department, Faculty of Pharmacy, Tanta University, Tanta 31111, Egypt; 4 Department of Pharmacology and Toxicology, Faculty of Pharmacy, Tanta University, Tanta 31111, Egypt; thanaa.elmasri@pharm.tanta.edu.eg; 5 Department of Anatomy, Faculty of Medicine, Tanta University, Tanta 31111, Egypt; walaa.elssaidi@med.tanta.edu.eg; 6 Department of Biochemistry, Faculty of Pharmacy, Al Azhar University, Cairo 11311, Egypt; 7 Health Services Directorate, Ministry of Defense, Riyadh 84428, Saudi Arabia; knalotaibi@yahoo.com

**Keywords:** GC/MS, histopathology, immunohistochemistry, biofilm, *Pseudomonas aeruginosa*

## Abstract

Lung diseases such as asthma, chronic obstructive pulmonary diseases, and pneumonia are causing many global health problems. The COVID-19 pandemic has directed the scientific community’s attention toward performing more research to explore novel therapeutic drugs for pulmonary diseases. Herein, gas chromatography coupled with mass spectrometry tentatively identified 44 compounds in frankincense ethanol extract (FEE). We investigated the antibacterial and antibiofilm effects of FEE against *Pseudomonas aeruginosa* bacteria, isolated from patients with respiratory infections. In addition, its in vitro immunomodulatory activity was explored by the detection of the gene expression of tumor necrosis factor-α (TNF-α), interleukin-6 (IL-6), nitric oxide synthase (iNOS), cycloxygenase-2 (COX-2), and nuclear factor kappa-B (NF-κB) in lipopolysaccharide (LPS)-induced peripheral blood mononuclear cells (PBMC). In addition, its anticancer activity against the A549 lung cancer cell line and human skin fibroblast (HSF) normal cell line was studied. Moreover, the in vivo lung protective potential of FEE was explored histologically and immunohistochemically in mice using a benzo(a)pyrene induced lung damage model. FEE exhibited antibacterial and antibiofilm activities besides the significant inhibition of gene expression of TNFα, IL-6, and NF-κB. FEE also exerted a cytotoxic effect against A549 cell line. Histological and immunohistochemical investigations with morphometric analysis of the mean area percentage and color intensity of positive TNF-α, COX-2, and NF-κB and Bcl-2 reactions revealed the lung protective activity of FEE. This study outlined the promising therapeutic activity of oleoresin obtained from *B. dalzielii* in the treatment of different pulmonary diseases.

## 1. Introduction

There are many pulmonary diseases of the upper and lower respiratory tract, such as chronic obstructive pulmonary diseases (COPD), asthma, bronchitis, pneumonia, and cystic fibrosis [1]. Despite the presence of many drugs for pulmonary diseases, these disorders cause a significant number of deaths every year worldwide, especially in the current time due to the pandemic disease COVID-19 [2,3]. In addition, many drugs currently used in the treatment of different pulmonary diseases can cause severe adverse effects. On the other hand, many plants are used for the treatment of asthma, cough, respiratory tract infections, and pneumonia all over the world [4]. Plants contain various safe secondary metabolites, such as phenol, alkaloids, resins, essential oils, and gum, which are responsible for their therapeutic effects [5,6,7,8,9].

Oleogum resin, or frankincense, is produced as a whitish exudate from the tree bark of different Boswellia species [10]. Oleoresin protects the trees against infections and pests. It has been used since ancient times for medicine and incense [11]. The present study investigated the frankincense (oleogum resin) of *Boswellia dalzielii* Hutch., family Burseraceae [12]. It is a tree, up to 13 m high, of the wooded savanna, which inhabits the northern Ivory Coast of Nigeria, Cameroun, and Ubangi-Shari. Frankincense oozes out from the tree bark as a milky fluid, which is gradually solidified by exposure to air. The gum part includes bassorin, while boswellic acids are found in the resin part, besides the volatile oil part with different terpenes and terpenoids which give the frankincense its characteristic odor. It was reported that frankincense had precious, ubiquitous, and important biological activities, such as anti-inflammatory, tumor suppressor [13], antimicrobial, antioxidant [14], and immunomodulatory [15] activities, as well as its ability to treat heart diseases and improve learning and memory in neurological diseases [16].

The present study investigated the effect of the oleoresin of *Boswellia dalzielii*, or frankincense ethanol extract (FEE), against Gram-negative *P. aeruginosa* clinical isolates and their biofilm. The opportunistic pathogen, *P. aeruginosa,* is a common etiological agent for many lung infections, particularly in patients suffering from impaired lung defenses such as patients with cystic fibrosis and COPD [17]. It commonly causes pulmonary diseases such as community or hospital acquired pneumonia, in addition to ventilator associated pneumonia [18]. Several researchers have screened different plant extracts for their antibiofilm activity against *P. aeruginosa* isolates, as they have an extraordinary ability to form biofilms [19,20].

The immune system (innate and adaptive) plays an important role in sustaining human life through protection against pathogenic organisms such as viruses, bacteria, and fungi. Thus, any disturbance in the functions of the immune system could lead to many diseases [21]. Immunomodulation causes either the induction or inhibition of any phase of the immunological response. Many immunomodulators have been obtained from diverse sources, such as plants, microorganisms, and fungi [22]. In the current study, the immunomodulatory activity of FEE on LPS induced PBMC was elucidated.

Benzo(a)pyrene or B(a)P, a polycyclic aromatic hydrocarbon, is one of the major constituents of tobacco smoke which has a main role in the induction of lung damage and carcinogenesis [23]. It results in many changes in the lung, leading to the development of many chronic disorders in the lung, including inflammation, edema, COPD, pulmonary fibrosis, and lung cancer [24,25].

The oleoresin of *Boswellia dalzielii* or frankincense ethanol extract (FEE) has not biologically investigated before, according to our knowledge. Therefore, we aimed to examine the antimicrobial and antibiofilm activities of FEE against the opportunistic pathogen, *Pseudomonas aeruginosa*, isolated from patients suffering from pulmonary diseases, which was performed for the first time. In addition, the anticancer activity on human nonsmall cell lung cancer cell line A549, and the immunomodulatory activity were studied in vitro. Moreover, the in vivo lung protective effect of FEE against induced lung damage by benzo(a)pyrene or B(a)P was elucidated in mice for the first time, to make a sufficient coverage about the ability of FEE to protect and treat the lung against different ailments in human beings.

## 2. Results

### 2.1. Gas Chromatography/Mass Spectroscopy (GC/MS) Analysis of FEE

**Figure 1:**
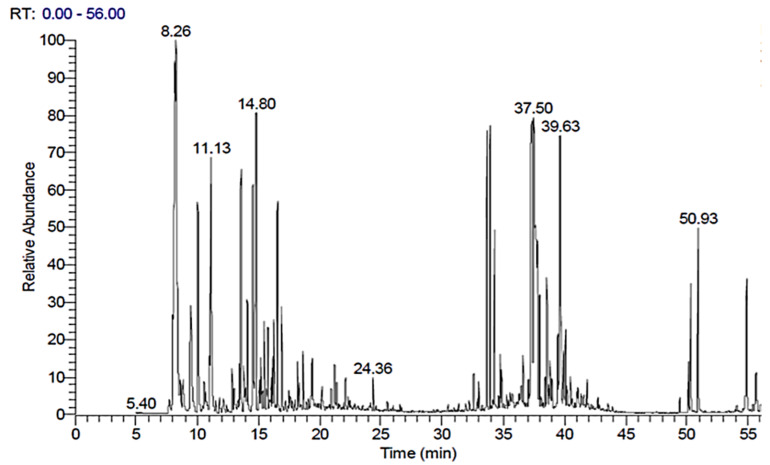
Mass spectrum of GC/MS analysis of frankincense ethanol extract (FEE).

**Figure 2:**
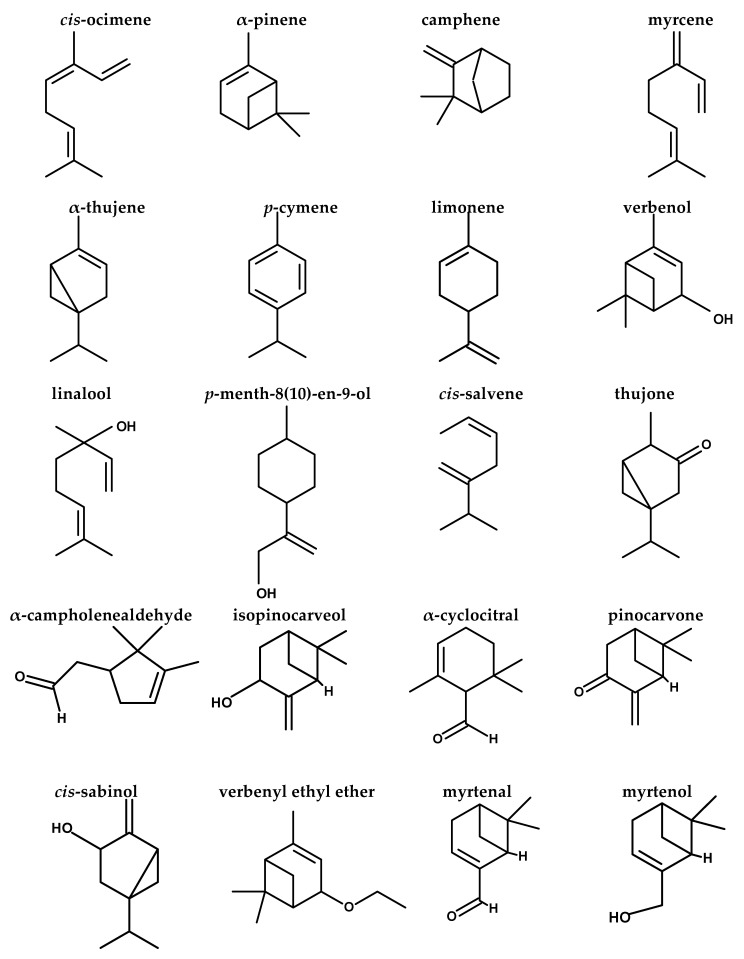

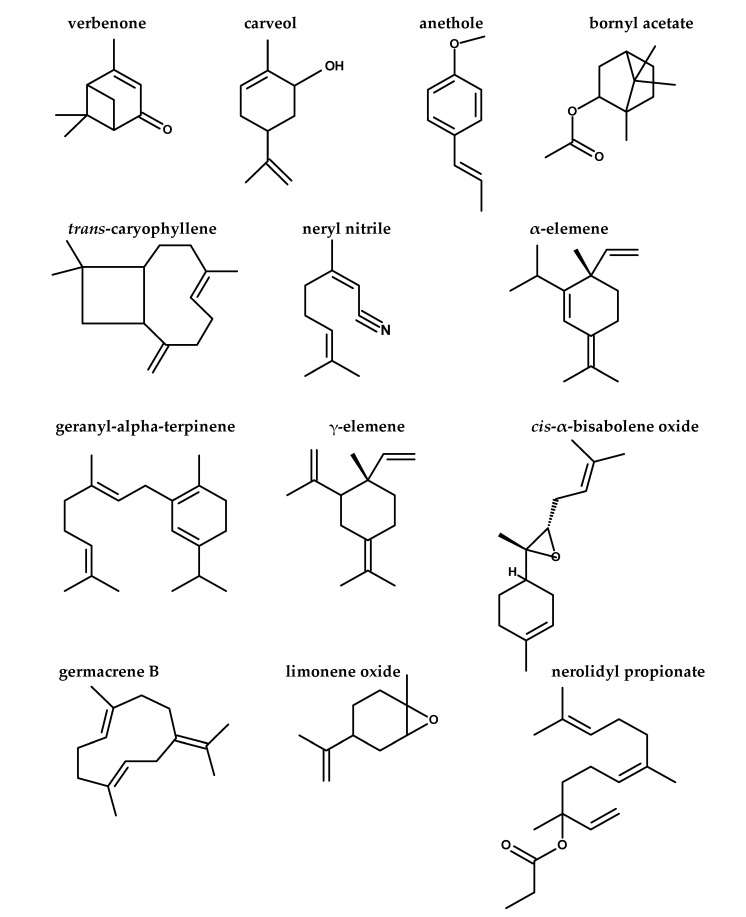
Structures of compounds identified by GC/MS analysis of FEE.

Table 1 and Figure 1 and Figure 2 demonstrated the different compounds established by mass spectrometry.

### 2.2. In Vitro Antibacterial and Antibiofilm Activities of FEE

#### 2.2.1. Antibacterial Activity

FEE exhibited antibacterial activity against the tested *Pseudomonas aeruginosa* clinical isolates with a range of minimum inhibitory concentration (MIC) values from 250 to 1000 µg/mL.

#### 2.2.2. Antibiofilm Activity

**Figure 3:**
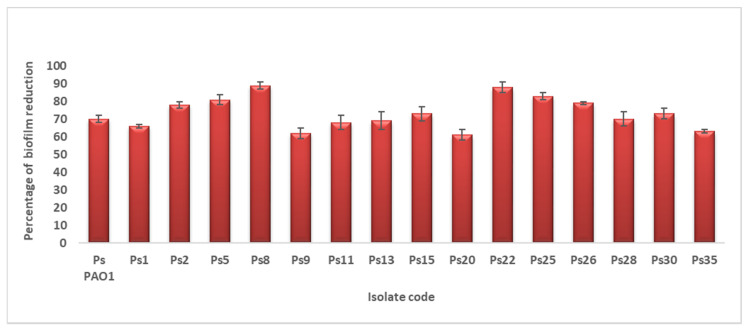
A chart showing the percentages of biofilm reduction by *P. aeruginosa* isolates after treatment with FEE.

**Figure 4:**
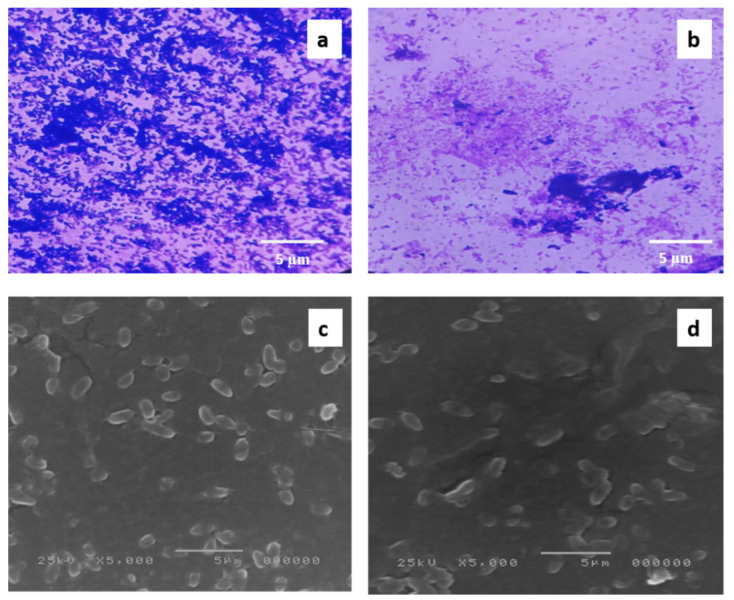
An illustrative example of the significant decline in the formation of biofilms by *P. aeruginosa* isolates after treatment with FEE (125 µg/mL). A bright field microscope was utilized to examine the formed biofilm: (**a**) before and (**b**) after treatment with FEE. SEM was utilized to observe the formed biofilm (**c**) before and (**d**) after treatment with FEE.

FEE displayed antibiofilm activity against the biofilm-forming *P. aeruginosa* isolates, as it caused a significant reduction in biofilm formation in 38.46% of the tested isolates, as shown in Figure 3, using *P. aeruginosa* PAO1 standard isolate, which is a biofilm-producing isolate, as a control. In addition, its impact on the biofilm morphology was studied using a bright field microscope, after staining of the biofilm with crystal violet, and scanning electron microscopes (SEM); a representative example is shown in Figure 4.

### 2.3. Immunomodulatory Activity of FEE

#### 2.3.1. Cell Viability Assay

**Figure 5:**
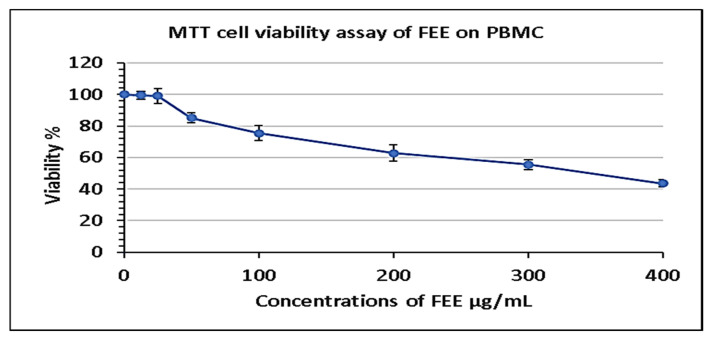
Cell viability assay of FEE on PBMC following 48 h incubation period. Different concentrations of FEE of 12.5, 25, 50, 100, 200, 300, and 400 µg/mL were used to treat PBMC, and MTT was utilized to determine the cell viability. IC_50_ was determined as the mean ± standard deviation (SD) of three independent experiments.

The effect of FEE, at concentrations of 12.5, 25, 50, 100, 200, 300 and 400 μg/mL, on the cell viability of PBMC was investigated using a 3-(4,5-dimethylthiazol-2-yl)-2,5-diphenyl-2*H*-tetrazolium bromide (MTT) assay. IC_50_ against peripheral blood mononuclear cells (PBMC) was determined at 343.49 ± 20.47, as shown in Figure 5.

#### 2.3.2. Assessment of FEE Effect on Genes Expression by Quantitative Real Time PCR (qRT-PCR)

**Figure 6:**
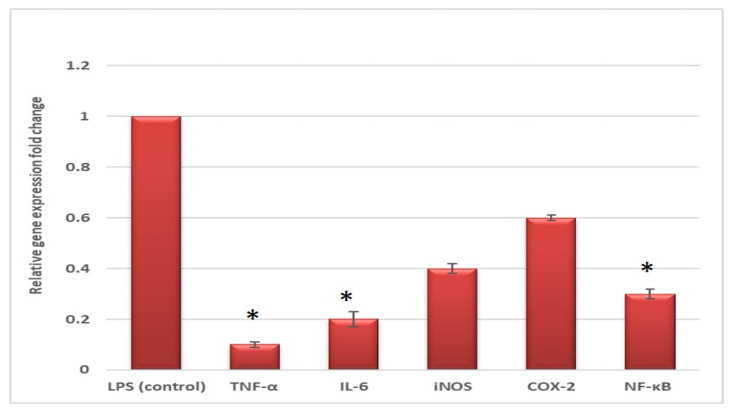
Effect of FEE on the expression of TNF-α, IL-6, iNOS, COX-2, and NF-κB in LPS induced PBMCs. * represents a significant reduction.

The mRNA expression of the bioactive molecules that are related to inflammation includes cytokines such as tumor necrosis factor-alpha (TNF-α) and interleukin-6 (IL-6), as well as enzymes such as nitric oxide synthase (iNOS) and cyclooxygenase-2 (COX-2), in addition to the transcription factor, nuclear factor kappa-B (NF-κB), was found to be induced in PBMC treated with lipopolysaccharides (LPS). Interestingly, FEE treatment, at 0.5 IC_50_, significantly attenuated the upregulation of the relative gene expression of TNF-α, IL-6, and NF-κB, compared to the nontreated LPS induced PBMCs, as shown in Figure 6.

### 2.4. In Vitro Anticancer Effect of FEE

**Figure 7:**
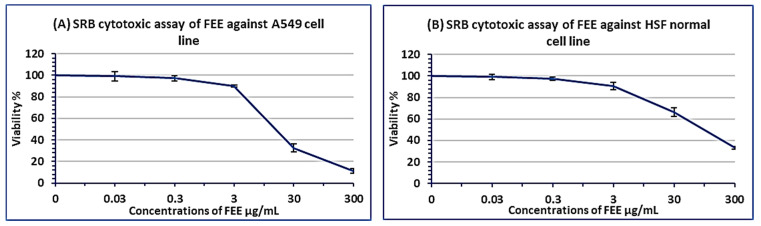
SRB cytotoxicity assay of FEE on A549 lung cancer cell line (**A**), and HSF human skin fibroblast normal cell line (**B**), after an incubation period of 72 h. Different concentrations of 0.03, 0.3, 3, 30 and 300 µg/mL of FEE were used. IC_50s_ values were expressed as mean ± SD of three performed assays.

Different doses of FEE (0.03, 0.3, 3, 30, 300 μg/mL) were tested by sulforhodamine B (SRB) cytotoxicity assay against A549 and HSF cell lines. IC_50_ against A549 lung cancer cell line and HSF normal cell line was determined at 21.8 ± 1.921 and 134.1 ± 2.442 μg/mL, respectively, as shown in Figure 7.

### 2.5. In Vivo Lung Protective Activity of FEE

#### 2.5.1. Effect of FEE on Lung Histological Characters

**Figure 8:**
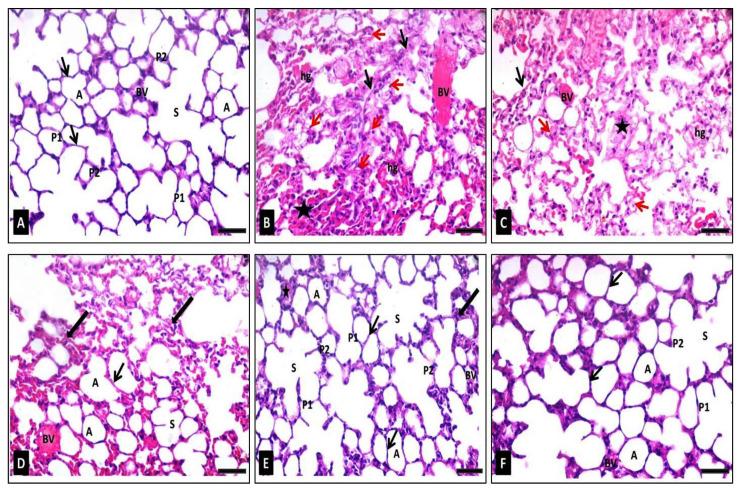
Photomicrographs of sections of the lung tissues of adult male mice stained with H&E showing: (**A**) Group I (control): alveolar sacs (s), alveoli with thin walls (arrows) formed of flat cells with densely stained nuclei type I pneumocytes (P1) and type II pneumocytes (P2) with large rounded nuclei, clear alveolar spaces (A), small thin-walled blood vessels (BV). (**B**) Group II (diseased): the lung architecture is disordered; some alveoli appear collapsed (star) with very thick alveolar walls (black arrows). Many alveolar epithelial lining cells show vacuolar cytoplasmic necrosis with pyknotic nuclei (red arrows). Severely congested blood vessels (BV) and interstitial hemorrhages (hg) could be seen. (**C**) Group III (diseased, 50 mg/kg FEE): moderate disorganization of alveoli; collapsed alveoli (star) with moderate thickening of the alveolar wall (black arrow) and many epithelial linings of the alveoli with pyknotic nuclei and vacuolated cytoplasm (red arrows). Moderately congested blood vessels (BV) and interstitial hemorrhages (hg) could be observed. (**D**) Group IV (diseased, 100 mg/kg FEE): some normal alveolar sacs (S) and alveoli (A) with thin walls (thin arrows). Notice, mild thickening of alveolar walls (thick arrows), congested blood vessels (BV), and minimal interstitial hemorrhages (hg). (**E**) Group V (diseased, 150 mg/kg FEE): the lung architecture is relatively near to the normal with alveolar sacs (S), clear alveolar spaces (A), thin-walled alveoli (thin arrows), normal type I pneumocytes (P1), as well as type II pneumocytes (P2) and small thin-walled blood vessels (BV). Few alveoli are collapsed (star) with minimal thick walls (thick arrow). (**F**) Group VI (150 mg/kg FEE only): the architecture of the lung is well organized. The alveolar sacs (S), alveolar spaces (A), the alveolar walls (arrows), type I pneumocytes (P1) and type II pneumocytes (P2) and blood vessels (BV) appear normal. [H&E ×400, scale bar = 50 μm].

The hematoxylin and eosin (H&E) stained sections of the lung specimens of the control group (group I) revealed the normal histological lung architecture: alveoli with thin walls, alveolar sacs, clear alveolar spaces, and thin-walled blood vessels. The epithelial lining of the alveoli was composed of attenuated squamous alveolar cells with densely flat nuclei (type I pneumocytes) and large alveolar cells with large, rounded nuclei (type II pneumocytes) (Figure 8A). Sections from group II (diseased) revealed a marked loss of lung architecture. The alveoli were distorted, and its epithelial lining depicted pyknotic nuclei and degenerated the vacuolization of its cytoplasm. Some alveoli were collapsed, with thickening of the alveolar septa. Severe congestion of the blood vessels and interstitial hemorrhage could be observed (Figure 8B). In the case of mice treated with 50 mg/kg b. w. of FEE; group III (diseased, 50 mg/kg FEE) showed a non-noticeable effect on the histopathological picture compared to group II (Figure 8C). Treatment with 100 mg/kg b. w. of FEE in group IV (diseased, 100 mg/kg FEE) resulted in partial improvement of the histological picture of the lung; alveolar spaces appeared clear with thin-walled alveoli. However, some distorted alveoli with partially thick walls and congested blood vessels could be detected (Figure 8D). Nevertheless, the mice sections treated with 150 mg of FEE, group V (diseased, 150 mg/kg FEE), showed obvious protection of its architecture to become close to the controls, however, few collapsed alveoli with thick walls could be detected (Figure 8E). The lung sections of mice administered with 150 mg/kg b. w. of FEE and not taken the single oral dose of B(a)P, group VI (150 mg/kg FEE only), showed normal histological appearance evidenced by normal thickening of most alveolar walls and normal epithelial lining, which indicated the drug safety at the maximum dose (Figure 8F).

#### 2.5.2. Immunohistochemical Studies

##### TNF-α Immunohistochemical Staining

**Figure 9:**
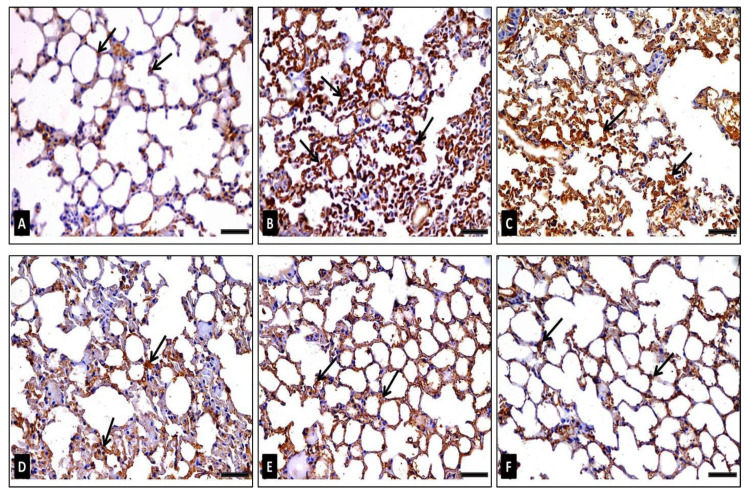
Photomicrographs representing TNF-α immunoexpression in lung sections of adult male mice displaying: (**A**) in group I (control): few cells having weak positive immunoreaction (arrows) represented by brown cytoplasmic coloration, (**B**) in group II (diseased): multiple cells with a very strong positive immunoreaction (arrows), (**C**) in group III (diseased, 50 mg/kg FEE): multiple cells with strong positive immunoreaction (arrows), (**D**) in group IV (diseased, 100 mg/kg FEE): certain cells with moderate positive immunoreaction, (**E**) in group V (diseased, 150 mg/kg FEE): few cells with weak positive immunoreaction and (**F**) in group VI (150 mg/kg FEE only): few cells with weak positive immunoreaction (arrows). [TNF-α immunostaining ×400, scale bar = 50 μm].

The control group immunostained sections (group I) exposed a few cells with cytoplasmic TNF-α positive immunohistochemical reaction expressed by brownish coloration (Figure 9A). However, the group II (diseased) sections showed frequent cells having a strong positive cytoplasmic TNF-α reaction (Figure 9B). At the same time, the group III (diseased, 50 mg/kg FEE) sections displayed multiple cells having a strong positive cytoplasmic TNF-α reaction (Figure 9C). However, the group IV (diseased, 100 mg/kg FEE) sections revealed certain cells having a moderately positive cytoplasmic TNF-α reaction (Figure 9D). However, the group V (diseased, 150 mg/kg FEE) sections depicted a nearly normal cytoplasmic TNF-α reaction (Figure 9E). The group VI (150 mg/kg FEE only) sections exhibited few cells with positive cytoplasmic TNF-α reaction (Figure 9F). Morphometric examination of the mean area percentage and the color strength of the positive TNF-α reaction exhibited a significant rise in these parameters in groups II and group III in comparison with the control group, nevertheless, in group IV, a significant decrease in both parameters was depicted, as compared to the diseased group II. In addition, a decrease was detected in these parameters in groups V and VI, which was nonsignificant in comparison with the control group and significant in comparison with the diseased group (Table 2).

##### COX-2 Immunohistochemical Staining

**Figure 10:**
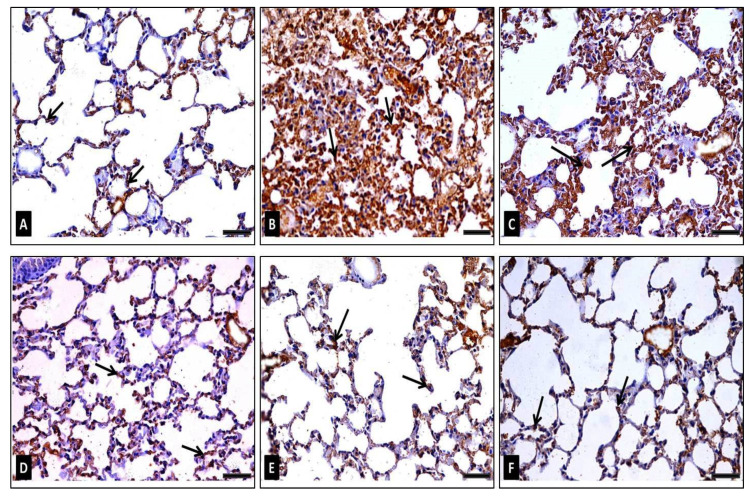
Photomicrographs representing COX-2 immunoexpression in lung sections of adult male mice displaying: (**A**) in group I (control): very weak positive immunoreactions in the cytoplasm of the lining alveolar epithelial cells (arrows), (**B**): in group II (diseased): very strong positive immunoreactions in the cytoplasm of the alveolar epithelium (arrows), (**C**) in group III (diseased, 50 mg/kg FEE): many cells having strong positive immunoreaction (arrows), (**D**) in group IV (diseased, 100 mg/kg FEE): certain cells exhibit moderate strong immunoreactions (arrows), (**E**) in group V (diseased, 150 mg/kg FEE): few cells with very weak positive cytoplasmic immunoreaction (arrows) and (**F**) in group VI (150 mg/kg FEE only): few cells with very weak positive immunoreaction (arrows). [COX-2 immunostaining ×400, scale bar = 50 μm].

Immunostained sections from the control group (group I) exhibited a low number of cells having a positive immunohistochemical reaction of cytoplasmic COX-2 depicted by a brownish coloration (Figure 10A). However, sections from group II (diseased) showed several cells having a strong positive COX-2 (Figure 10B). On the other hand, group III (diseased, 50 mg/kg FEE) sections revealed many cells with a moderately positive COX-2 reaction (Figure 10C). Nevertheless, group IV (diseased, 100 mg/kg FEE) sections showed some cells having a positive COX-2 reaction (Figure 10D). Sections from group V (diseased, 150 mg/kg FEE) exhibited a near normal COX-2 reaction (Figure 10E). However, sections of group VI (150 mg/kg FEE only) showed few cells with a positive cytoplasmic COX-2 reaction, similar to the control (Figure 10F). Morphometric analysis of the mean area percentage and the color strength of the positive COX-2 reaction represented a significant increase in these parameters in group II and group III, in comparison with the control group. While group IV revealed a significant decrease in both parameters compared to the control group. A nonsignificant alteration was detected in these parameters in groups V and VI in comparison with the control group, though a significant alteration was observed in comparison with the diseased group (group II) (Table 2).

##### NF-κB (P65) Antigen Immunohistochemical Staining

**Figure 11:**
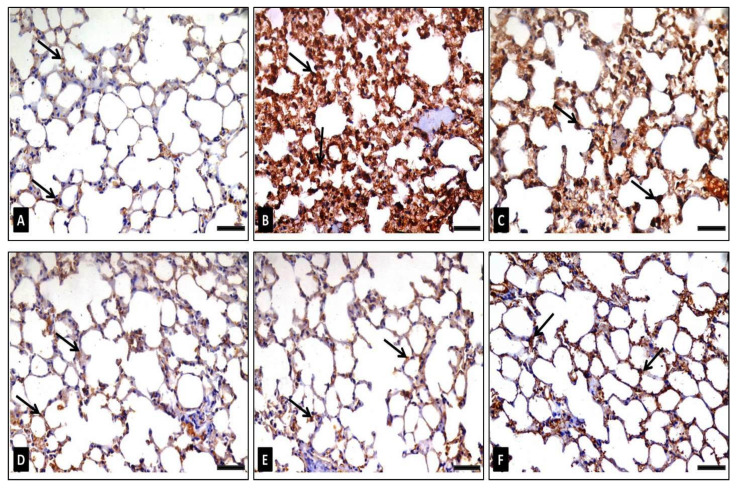
Photomicrographs representing NF-κB (P65) immune stained lung sections of adult male mice exhibiting: (**A**) in group I (control): weak positive brown nuclear immunoreaction for NF-κB in a very low number of cells, (**B**) in group II (diseased): very strong positive NF-κB (P65) nuclear immunoreaction for in a large number of cells (arrows), (**C**) in group III (diseased, 50 mg/kg FEE): moderate positive NF-κB (P65) nuclear immunoreaction in certain cells (arrows), (**D**) in group IV (diseased, 100 mg/kg FEE): moderate positive NF-κB (P65) nuclear immunoreaction in a low number of cells (arrows), (**E**) in group V (diseased, 150 mg/kg FEE): weak positive nuclear NF-κB (P65) immunoreaction in a very low number of cells (arrows) and (**F**) in group VI (150 mg/kg only): weak positive nuclear NF-κB (P65) immunoreaction in a low number of cells (arrows). [NF-κB immunostaining×400, scale bar = 50 μm].

In the control group NF-κB-immunostained sections (group I), a small number of cells displayed a weak positive brown nuclear with or without perinuclear cytoplasmic NF-κB immunoreactions (Figure 11A). Sections of group II (diseased) had many cells having a very strong positive nuclear with or without perinuclear cytoplasmic NF-κB immunoreaction (Figure 11B). At the same time, group III (diseased, 50 mg/kg FEE) immunostained sections exhibited only a moderate positive nuclear, with or without perinuclear cytoplasmic NF-κB immunoreaction in certain cells (Figure 11C). In group IV (diseased, 100 mg/kg FEE), sections showed certain cells with a positive nuclear, with or without perinuclear cytoplasmic NF-κB immunoreaction (Figure 11D). Sections from group V (diseased, 150 mg/kg FEE) exhibited a near normal NF-κB reaction (Figure 11E). However, sections of group VI (150 mg/kg FEE only) showed few cells with a weak positive nuclear, with or without a perinuclear cytoplasmic NF-κB immunoreaction, similar to the control (Figure 11F). Morphometric analysis of both the mean area percentage and the color strength of the positive NF-κB reaction unveiled a significant rise in these parameters in group II and group III in comparison with the control group, whereas group IV displayed a significant reduction in these parameters in comparison with the control. Groups V and VI displayed a significant lessening in these parameters in comparison with the diseased group (group II) and a nonsignificant alteration was detected according to control (group I) (Table 2).

##### B-Cell Lymphoma-2 (Bcl-2) Immunohistochemical Staining

**Figure 12:**
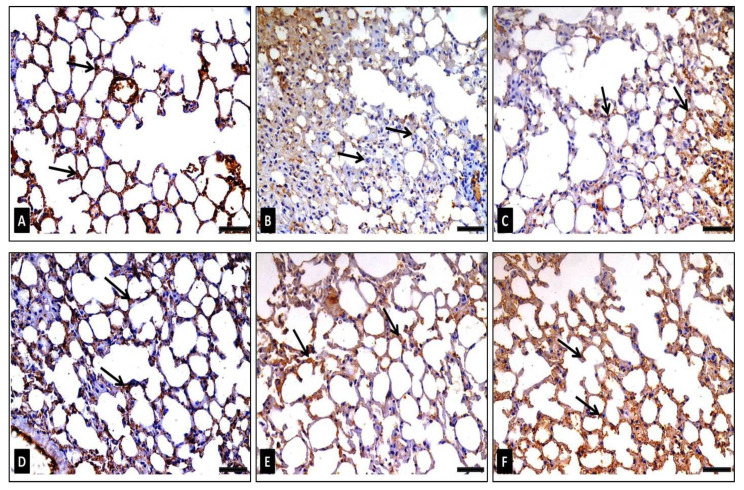
Photomicrographs of Bcl-2 immuno-expression in adult male lung sections revealing: (**A**) in group I (control): many cells having strong positive immunoreaction (arrows) represented by the brown color of the cytoplasm, (**B**) in group II (diseased): very weak immunoreaction (arrows), (**C**) in group III (diseased, 50 mg/kg FEE): few cells with positive Bcl-2 immunoreaction (arrows), (**D**) in group IV (diseased, 100 mg/kg FEE): certain cells with strong positive immunoreaction, (**E**) in group V (diseased, 150 mg/kg FEE): multiple cells with strong positive immunoreaction and (**F**) in group VI (150 mg/kg FEE only): frequent cells with strong positive immunoreaction (arrows). [Bcl-2 immunostaining ×400, scale bar = 50 μm].

The Bcl-2 immuno-stained sections of the control group (group I) displayed a strong positive cytoplasmic Bcl-2 immunoreaction (Figure 12A). Sections of group II (diseased) showed a very weak Bcl-2-immunoreaction in its cytoplasm (Figure 12B). Group III (diseased, 50 mg/kg FEE) revealed a mild positive Bcl-2-immunoreaction (Figure 12C). A moderate immunostaining reaction for Bcl-2 was detected in group IV (diseased, 100 mg/kg FEE) (Figure 12D). Nevertheless, sections from group V (diseased, 150 mg/kg FEE) depicted a stronger Bcl-2 immunostaining (Figure 12E). Sections from group VI (150 mg/kg FEE only) revealed a strong positive cytoplasmic Bcl-2 reaction (Figure 12F). Morphometric examination of the mean area percentage and the color strength of positive Bcl-2 immunoreaction revealed a significant reduction in group II and group III, in comparison with the control group. Meanwhile, a significant increase was detected in groups IV, V and VI, in comparison with the diseased group. No statistically significant alteration between group V and the control group was revealed (Table 2).

## 3. Discussion

There is a significant need for the development of novel therapeutic compounds for lung disorders, as many of them could lead to lung failure and subsequent death. The recent pandemic disease, COVID-19, has focused the world’s attention on the importance of the presence of new alternatives to the currently present drugs, to be able to treat the nonresponding cases. Thus, we decided to explore the antibacterial and antibiofilm activities of frankincense ethanol extract (FEE) against *P. aeruginosa* clinical isolates from patients suffering from respiratory tract infections. The linkage between inflammation induced cancer and pathogenic infectious organisms was confirmed. The bacterial pathogens cause chronic inflammatory conditions characterized by irritation and tissue damage, which lead to malignant changes (cancers) [26]. Therefore, experimental studies of its in vitro immunomodulatory and anti-lung cancer effects were performed. Moreover, the in vivo lung protective influence of FEE on the lung damage resulting from B(a)P in mice was carried out for the first time.

Different Boswellia trees are the source of frankincense oleogum resin, which contains a complex mixture of compounds, e.g., essential oils of monoterpenes, diterpenes, and others, as well as penta- and tetracyclic terpenoids, e.g., α- and *β*-boswellic acids (BA), acetyl-α-boswellic acid (ABA), 11-keto-*β*-boswellic acid and 3-*O*-acetyl-11-keto-*β*- boswellic acid (AKBA) [27], in addition to lupeolic acids, incensole, cembrenes and triterpenediol [13]. In addition, it contains other compounds, such as polyphenols, alkaloids, saponins, and polysaccharides [28]. The reported remarkable biological activities of frankincense oleogum resin are mostly attributed to the pentacyclic boswellic acids of the resin especially AKBA [16], and the monoterpenes of the essential oils of Boswellia species [15]. The active parts are the oil and resin as previously mentioned, therefore in this study, they were extracted with ethanol to obtain FEE from *B. dalzielii*. Little attention has been paid to the study of the biological effects of the oleoresin of *B. dalzielii*, besides, it was reported that the oil content varies between different Boswellia species in different countries according to the environment’s climate and harvest conditions. [29].

The dissimilar commercial varieties of frankincense could be distinguished by the GC/MS profile of the essential oil, which is used as a chemotaxonomical marker [30]. The current study investigated FEE phytochemically by GC/MS analysis, which revealed the identity of 44 different terpenes, (tentatively) of terpenoids and other compounds. The major compounds were limonene oxide, *cis*-ocimene, α-pinene, α-cyclocitral, α-elemene, limonene, neryl nitrile, *p*-menth-8(10)-en-9-ol, α-thujene, isopinocarveol, verbenone, myrcene, and *p*-cymene. A previous study [10] concerning the essential oil of the oleoresin of *B. dalzielii* trees from northern Nigeria revealed that the major terpenes were α-pinene, limonene, α- thujene, and myrcene with a lower level of *p*-cymene. It was noticed that the essential oil contains mostly monoterpenes and nearly lacks sesquiterpenes. The previous study confirmed the oleoresin biological source, as the volatile oil composition of frankincense is consistent with the reported data for the oleogum resin obtained from *B. dalzielii* [10]. The essential oil possessed immunomodulatory [15], anticancer [31], and antimicrobial effects [14]. In addition, it was found that the volatile oil from the frankincense oleoresin of *B. carterii* exerted antimicrobial activities against various microorganisms [32]. The α- and *β*- boswellic acids, as well as their derivatives, exerted outstanding anti-inflammatory effects, in addition to anticancer, immunomodulatory, and antimicrobial effects [16]. Acetyl-11-keto-*β*-boswellic acid (AKBA), a highly active component of the therapeutic frankincense from *Boswellia* species, is a pentacyclic terpenoid effective against many inflammatory diseases, including asthma, arthritis, Crohn’s disease, chronic and ulcerative colitis [16]. In a previous study, it was found that BAs influence the cellular defense system via their interaction with the release of cytokines. Boswellic acids suppress activation of NF-κB, resulting in a decrease in proinflammatory cytokine production [33].

Owing to the capability of *P. aeruginosa* to resist several antibiotics and its biofilm-forming ability, developing new nonantibiotic antimicrobials is a priority. This study revealed that FEE had MIC values that ranged from 250 to 1000 µg/mL against the tested isolates. In addition, it showed antibiofilm activity against the biofilm-forming *P. aeruginosa* isolates. Biofilm is a bacterial aggregation enclosed in a matrix of extracellular polymeric substances. It is one of the bacterial strategies to survive in the changes of the living conditions such as fluctuation in temperature and availability of nutrients [34].

Macrophages are considered the main cells of the innate immune system, as they afford a first line defense for protecting the body from different infections. LPS, a main constituent of the Gram-negative bacterial outer membrane, is a strong activator for macrophages [35]. When macrophages are activated in response to a stimulus such as LPS, they produce inflammatory cytokines such as TNF-α, IL-6, in addition to inflammatory mediators such as nitric oxide (NO) and prostaglandins (PGs). NO is produced from the activity of iNOS on the amino acid, L-arginine, and PGs are produced by the action of COX-2 enzyme on arachidonic acid [36]. Activated macrophages overexpress these enzymes (iNOS and COX-2), resulting in the production of vast amounts of NO and PGs. Besides, NF-κB is a transcription factor that induces the proinflammatory genes, to produce large amounts of the proinflammatory mediators such as iNOS and COX-2 in LPS induced macrophages [37]. All these bioactive compounds have a host defensive action during the inflammatory process. Overexpression of such molecules could be detrimental to the tissues as it could result in many autoimmune diseases that could lead to death. Thus, inhibition of such interactions offers a new therapeutic strategy for the treatment of infectious diseases, e. g., lungs infected with *P. aeruginosa* [38] as well as against many autoimmune diseases [39]. The use of immunomodulators from natural products, such as antimicrobial drugs, could solve the problem of bacterial resistance against traditional antibiotics, because they do not deal with microorganisms directly. Along with this, they could help the immunocompromised patients, who suffer from inactivity of antimicrobials to treat their infections [38].

Herein, we determined the relative gene expression of TNF-α, IL-6, iNOS, COX-2, and NF-κB in LPS induced PBMC after treatment with FEE. FEE exhibited a significant decrease in the upregulation of the relative expression of the tested genes of TNF-α, IL-6, and NF-κB when compared to the untreated LPS-induced PBMCs. Many researchers have investigated the impact of plant extracts on the relative gene expression of inflammation-related enzymes and cytokines [40,41].

It was reported that the direct link between cancer and an infectious disease due to aberrant pathogenic microbial colonization occurred in about 20% of all cancer cases [42]. We should consider the possibility that patients who suffer from COPD and secondary infections caused by *P. aeruginosa* may have lung cancer. Therefore, in vitro anti-lung cancer activity of FEE was investigated. SRB cell viability assay of FEE against A549 lung cancer cell line and HSF normal cell line was conducted. IC_50_ of FEE was 21.8 ± 1.921 and 134.1 ± 2.442 μg/mL, respectively. This indicated a possible anti-lung cancer effect of FEE, with a selectivity index of 6.15. The linkage between chronic inflammatory diseases and cancer was established [43]. The anticancer effect of frankincense was reported to be due to modification in signaling transduction, resulting in the inhibition of proliferation and metastasis [13].

In this study, short-term exposure of the mice lungs to B(a)P was utilized as a model to induce lung damage to study the potential lung protective effect of FEE by histological assessment of the lung tissues and measuring inflammatory and apoptosis markers such as TNF-α, COX-2, NF-κB, and Bcl-2. It was found that the FEE dose of 150 mg/kg b.w. showed the best effect in comparison with the control group. Concerning safety, the maximum dose of FEE of group VI (150 mg/kg FEE only), without taking the single oral dose of B(a)P, showed normal lung histology with normal thickening of alveolar walls and normal epithelial lining.

The histology of mice lung sections was affected differently according to the treatment type. Mice sections were normal in control group I, while the diseased group II showed severe lung distortion. The treated groups IV and V with FEE 100 and 150 mg/kg b.w., respectively, showed better lung architecture, especially group V. Normal alveolar sacs, clear alveolar spaces, thin-walled alveoli, normal type I pneumocytes and type II pneumocytes, and small thin-walled blood vessels, were noticed in group V.

The immune stained lung sections of mice of groups I, VI, V, and VI exhibited a normal reaction of few cells with positive cytoplasmic TNF-α, Cox-2, NF-κB reactions. On the other hand, groups II and III showed many cells with strong and moderate positive cytoplasmic TNF-α, Cox-2, NF-κB reactions, respectively, expressed by a brownish color. Morphometric analysis revealed that treatment with 150 mg/kg b.w., with or without administration of a single dose of B(a)P, in groups V and VI reduced the mean area percentage and the color strength of positive TNF-α, Cox-2, NF-κB reactions significantly in comparison to the diseased group II. On the other hand, treatment with 100 mg/kg b.w. (group IV, diseased, 100 mg/kg FEE) decreased both mean area percentage and the color intensity of positive TNF-α, Cox-2, NF-κB reactions significantly, compared with the control and the diseased groups. Mice of group III (diseased, 50 mg/kg FEE) showed multiple cells with strong positive cytoplasmic TNF-α Cox-2, NF-κB reactions. Bcl-2 immunohistochemical staining of lung sections of mice demonstrated a strong positive reaction in groups I, V, and VI, while it displayed a moderate reaction in group IV (diseased, 100 mg/kg FEE). Groups II (diseased) and III (diseased, 50 mg/kg FEE) showed weak to mild positive Bcl-2 immunoreaction. Morphometric examination of the mean area percentage and the color strength of positive Bcl-2 immunoreaction exhibited a highly significant reduction in group II (diseased) and group III (diseased, 50 mg/kg FEE) in comparison with the control group. Meanwhile, a significant increase was noticed in group IV (diseased, 100 mg/kg FEE) in comparison with the diseased group. Groups V and VI showed no statistically significant difference according to the control group, but they displayed a significant increase when compared to the diseased group II. Bcl-2 is an anti-apoptotic protein that is known to be an oncogene as when its level is increased it stops the mitochondrial discharge of cytochrome C resulting in inhibition of the apoptosis process [44].

## 4. Materials and Methods

### 4.1. The Utilized Chemicals

All chemicals were purchased from Sigma-Aldrich, USA. Anti-TNF-α antibody (ab6671), anti-COX-2 antibody (ab15191), anti-NF-κB p65 antibody (ab17742), and anti-Bcl-2 antibody were purchased from Abcam corporation (Waltham, MA, USA).

### 4.2. Preparation of Frankincense Ethanol Extract

Frankincense oleogum resin (200 g) was purchased in May 2019 from a herbal store located at Al Farwaniyah in Kuwait. It was obtained from trees of *B. dalzielii* Hutch. growing in Nigeria. The tree description was consistent with that of *B. dalzielii* Hutch. [45]. A voucher specimen (PG-U-00416) was deposited at the Herbarium of Pharmacognosy Department, Faculty of Pharmacy, Tanta University. It was powdered and macerated in ethanol 100% (300 mL × 3 times) at RT. Evaporation of combined ethanol extract was performed with a rotary evaporator at 40 °C under vacuum to have 8.5 g of yellow semisolid mass.

### 4.3. GC/MS Data Analysis

Thermo Scientific, Trace GC Ultra / ISQ Single Quadrupole MS for GC/MS analysis of frankincense ethanol extract. The column type was TG-5MS fused silica capillary column (30 m, 0.251 mm, 0.1 mm film thickness, Waltham, MA, USA). The ionization mode, which was used for GC/MS detection, was an electron ionization system of 70 eV ionization energy. The carrier gas was helium, which was used at a constant flow rate of 1mL/min. The temperature of the injector and MS transfer line was set at 280 °C. The temperature programming of the oven was set at an initial temperature of 40 °C for 3 min, then was increased to a final temperature of 280 °C by an increasing rate of 5 °C/min and held for 5 min.

The percent relative peak area was used for the quantitative determination of the identified components. The characterization of compounds was performed tentatively, based on the comparison of their relative retention times and mass spectra with those of the NIST, WILEY library data of the GC/MS system.

### 4.4. In Vitro Antibacterial and Antibiofilm Activities of FEE

#### 4.4.1. Collection and Identification of *P. aeruginosa* Clinical Isolates

Thirty-nine *P. aeruginosa* isolates were collected from tracheal aspirate, bronchoalveolar lavage, and sputum samples from patients suffering from cystic fibrosis and COPD, as previously described [46]. Isolates were identified by Gram stain and biochemical tests [47]. *Pseudomonas aeruginosa* ATCC 27853 was used as a standard strain.

#### 4.4.2. Screening of the Antibacterial Activity

The antibacterial activity of FEE was tested against *P. aeruginosa* clinical isolates using the agar well diffusion method [48]. The surfaces of tryptic soya agar (TSA) plates were flooded with 100 µL of bacterial suspension of each isolate. Wells of 5 mm diameter were filled with 100 µL of FEE (1000 µg/mL). The plates were then incubated overnight at 37 °C. The diameters of the inhibition zones were determined in mm.

#### 4.4.3. MIC Determination

The FEE MICs were determined by the broth microdilution method as previously described [49].

#### 4.4.4. Antibiofilm Activity

This was performed according to [50], before and after treatment with 0.5 MIC values of FEE (with a range from 125 to 500 µg/mL). In brief, after inoculation of 100 µL of *P. aeruginosa* suspensions into a 96-well microtitration plate at 37 °C for 24 h, the cells that did not adhere to the bottom of the wells were gently removed by washing with phosphate buffered saline (PBS) three times and then were left for drying for 1 h. Crystal violet (0.1%) was used to stain the formed biofilms and the samples were thoroughly washed using distilled water. Finally, 125 µL acetic acid (30%) was added to each well and left for 15 min and optical density (OD) values were measured at 492 nm using ELISA Auto Reader (Sunrise Tecan, Austria). The biofilm reduction percentage was detected by the equation:

$$Biofilm\;inhibition\;percentage=100\times \frac{OD\;before\;treatment-OD\;after\;treatment}{OD\;before\;treatment}$$

#### 4.4.5. Effect of FEE on Biofilm Morphology Using Bright Field and SEM

Biofilm morphology was investigated before and after treatment with 0.5 MIC values of FEE (ranged from 125 to 500 µg/mL) by a bright field microscope and SEM. After allowing each biofilm-forming bacteria to grow on 2 glass slides overnight, the first glass slide was stained with crystal violet for examination by a bright field microscope according to Attallah, N.G.M et al. [51]. Besides, the second glass slide was prepared according to the method mentioned in [52] for examination by SEM.

### 4.5. Immunomodulatory Effect of FEE

#### 4.5.1. Isolation of PBMCs

These were attained from a healthy human subject. They were isolated from the human blood by Ficoll density gradient centrifugation and then cells were cultured in Roswell Park Memorial Institute (RPMI 1640) medium supplemented with 10% fetal bovine serum, 2 mM L-glutamine, and 1% penicillin-streptomycin solution in flat bottom 6-well plates and then incubated in a humidified atmosphere of 5% CO_2_ at 37 °C for 24 h.

#### 4.5.2. MTT Cell Viability Test

Toxicity of FEE, at concentrations 12.5, 25, 50, 100, 200, 300, and 400 µg/mL, was assessed on PBMC using the MTT cell viability test. Mean inhibitory concentration (IC_50_) of FEE on PBMC was calculated, and half IC_50_ was utilized to explore the immunological responses in LPS induced PBMC, as previously described [53]. PBMC were regularly cultured in Dulbecco’s modified Eagle medium (DMEM). This medium contained glucose, L-glutamine, 10% (*v*/*v*) fetal bovine serum, and 1% (*v*/*v*) penicillin/streptomycin/amphotericin B. They were incubated at 37 °C in 5% CO_2_ [54]. The cells were then seeded (at a concentration of 1 × 10^4^ cells/mL) into 96-well cell culture plates and incubated for 24 h in DMEM with the previously mentioned supplements till reaching the exponential growth. They were incubated for 48 h with different concentrations of FEE, after that the medium was removed and 5 mg/mL of MTT reagent was added to each plate and incubated for three to four hours. An ELISA microplate reader was used to assess the absorbance at 630 nm after dissolving formazan crystals in 100 μL acidified isopropanol [55].

#### 4.5.3. qRT-PCR

The quantitative detection of the gene expression of TNF-α, IL-6, iNOS, COX-2, and NF-κB in LPS induced PBMC model after treatment with FEE was performed to examine the in vitro immunomodulatory impact of FEE on human macrophages. In brief, 2 × 10^6^ cells/mL of PBMCs in RPMI 1640 medium were seeded into 6-well plates with a flat bottom for 24 h. Then, 100 μL of *Escherichia coli* LPS (20 ng/mL) was added to the cells for 24 h in the presence and absence of FEE (with a concentration of 0.5 IC_50_) [40]. The effect of FEE on the gene expressions of TNF-α, IL-6, iNOS, COX-2, and NF-κB was evaluated using qRT-PCR and the utilized primers are shown in Table S1. After extraction of total RNA from PBMCs using RNeasy mini kit (Qiagen, Hilden, Germany), it was retrotranscribed to complementary DNA (cDNA) using SensiFAST™ cDNA kit (Bioline, London, UK) and GAPDH was utilized as a housekeeping gene. SensiFAST™ SYBR green PCR master mix (Bioline, London, UK) was used. The fold change in the gene expression relative to the control (untreated cells) was detected using the 2^−ΔΔCT^ method [50].

### 4.6. In Vitro Anti-Lung Cancer Effect of FEE (SRB Assay)

Nawah Scientific Inc., (Mokatam, Cairo, Egypt) provided A549 (lung cancer) and HSF (human skin fibroblast) cell lines. In humidified, 5% CO_2_ atmosphere at 37 °C. The SRB cell viability assay was performed. Aliquots of 100 μL cell suspension (5 × 10^3^ cells) were cultured in 96-well plates for 24 h. Other aliquots of 100 μL media containing different concentrations (0.03, 0.3, 3, 30, 300 µg/mL) of FEE were put to the cells. The drug treatment lasted for 72 h, after that, the cells were fixed by exchanging the medium with 150 μL of 10% trichloroacetic acid (TCA) and the incubation process was performed for one hour at the refrigerator. The TCA solution was excluded from the equation, and the cells were washed five times with distilled water. 70 μL SRB solution (0.4% *w*/*v*) was included in aliquots and left at room temperature for 10 min in a dark place. Plates were washed with 1% acetic acid and left to dry in the air overnight. Then, the proteinbound SRB stain was dissolved by 150 μL of TRIS (10 mM); the OD values were determined at 540 nm using Omega ELISA reader (Ortenberg, Germany) [45].

### 4.7. In Vivo Lung-Protective Effect of FEE

#### 4.7.1. Animals

Healthy, male Swiss albino mice 10–12 weeks old that weighed 28–35 g were utilized for this study. The protocol of the experiment was approved and accredited by the Research Ethical Committee of the Faculty of Pharmacy, Tanta University, Egypt (FPTU-REC, 105/2020/321). Mice stayed in rooms with good ventilation, and the temperature was kept at room temperature with a 12 h light/dark cycle. Mice were housed in plastic (polypropylene) cages in groups of six mice per cage in the animal house of the Faculty of Medicine, Tanta University. They were provided with free access to the standard laboratory water and feed.

#### 4.7.2. Induction of Lung Damage in Mice by Benzo (a) Pyrene

Groups of 6 mice each were administered orally with different solutions of 30 μL or 0.03 mL per mouse as follows: Group I or control group was administered 70% ethanol daily for 7 days, Group II or diseased group took 70% ethanol daily for 7 days, then a single oral dose of 0.4 mL of B(a)P in corn oil (125 mg/kg b. w.) on 7th day only, Groups III, IV, and V were treated with 50, 100, 150 mg/kg b.w. of FEE in 70% ethanol, respectively, daily for 7 days, then a single oral dose of 0.4 mL of B(a)P in corn oil (125 mg/kg b. w.) on the 7th day, Group VI was administered 150 mg/kg b. w. of FEE in 70% ethanol daily to study the safety of this drug. Following 24 h, the studied animals were anesthetized using mild ether anesthesia and then sacrificed by cervical dislocation technique. Samples of lung tissues were acquired for histological investigations and immunohistochemistry studies.

#### 4.7.3. Preparation of the Specimens for Histological Examination

Lung specimens were immediately extracted and fixed in 10% buffered formalin. Lung specimens were processed and embedded in paraffin blocks and stained with H&E as previously described [49].

#### 4.7.4. Specimen Preparation for Immunohistochemistry Investigations

Serial 5 μm sections were deparaffinized and hydrated, then trypsin solution was added to the sections for 15 min for retrieval of the antigens. Hydrogen peroxide solution in methanol was used for blocking the endogenous peroxidase activity. Then, the sections were thoroughly washed with PBS and incubated in normal goat serum for blocking any unspecific binding. The sections were then stained using the primary antibodies for 12 h and they were rinsed again using PBS and allowed to be left with the biotinylated secondary antibodies for 1 h. After that, they were allowed to be left with peroxidase conjugated streptavidin for 1 h. The color reaction was attained by allowing the sections to be incubated with diaminobenzidine (DAB) and hydrogen peroxide for 5 min using hematoxylin as a counterstain. Negative controls were prepared by excluding the primary antibodies [50]. The reaction appears as brown cytoplasmic deposits in the case of anti-TNF-α and anti-COX-2. NF-κB immunostained lung sections were regarded as positive when showing obvious brown nuclear with or without perinuclear cytoplasmic coloration. Cells were considered positive when showing brown cytoplasmic precipitation with Bcl-2.

#### 4.7.5. Morphometrical Examination

The sections were examined using Leica light microscope (DM500, AG, 9435 Heerbrugg, Swizterland) and photographed using a digital camera (ICC50, Wild Heerbrugg, Swizterland) fixed to the microscope using Image J software, version 1.48v, (Bethesda, Maryland, MD, USA) for image analysis. Ten diverse nonoverlapping fields from the slides were inspected using a magnification of 400 for quantitative estimation of the percentage of the mean area and color strength of TNF-α, COX-2, NF-κB, and Bcl-2 positive immunohistochemical reactions.

### 4.8. Statistical Analysis

The results were revealed as mean ± SD. The differences between the different tested groups were established using a one-way analysis of variance (ANOVA), followed by a post hoc test (Tukey). *p* values < 0.05 were regarded to be statistically significant. All statistics were carried out using SPSS, version 23.0 (IBM Corp, Armonk, NY, USA).

## 5. Conclusions

This study was carried out to reveal some of the active components of the frankincense ethanol extract (FEE) of *B. dalzielii*. by the GC/MS profile. FEE could treat lungs against various disorders, e. g., infectious disease, cancer, and lung damage induced by B(a)P. It showed antibacterial and antibiofilm effects against *Pseudomonas aeruginosa* clinical isolates from patients with diseased lungs. FEE exhibited a significant immunomodulatory downregulation effect on TNF-α, IL-6, and NF-κB gene expression in LPS induced PBMC. This could be a promising strategy for new antimicrobial drugs. FEE as an immunomodulator may solve the problem of bacterial resistance. In addition, it may expand the treatment options for immunocompromised patients suffering from poor traditional antimicrobials effect. Moreover, the anti-inflammatory effect of FEE by reducing the NF-κB, COX-2, and TNF-α immunoreactions in mice, with B(a)P- induced lung damage, could be considered in the prevention of inflammation induced cancer of the lung.

## Figures and Tables

**Table 1:** GC/MS analysis of frankincense ethanol extract.

No.	R_t_ (min.)	Peak Area %	Compound	Library
1	8.16	6.38	*cis*-Ocimene	Wiley
2	8.26	5.99	α-Pinene	Wiley
3	8.62	0.65	Camphene	Wiley
4	9.46	2.85	Myrcene	Wiley
5	10.03	3.50	α-Thujene	Wiley
6	11.02	0.74	*p*-Cymene	Wiley
7	11.13	4.26	(+)-Limonene	Wiley
8	12.83	0.62	Verbenol	Wiley
9	13.42	0.43	Linalool	Wiley
10	13.50	3.63	*p*-Menth-8(10)-en-9-ol	Mainlib and Wiley
11	13.57	0.61	*cis*-Salvene	Wiley
12	13.79	0.60	Thujone	Wiley
13	14.09	1.42	α-Campholenealdehyde	Wiley
14	14.53	3.15	Isopinocarveol	Wiley
15	14.80	5.80	α-Cyclocitral	Wiley
16	15.18	0.59	Pinocarvone	Mainlib and Wiley
17	15.45	1.08	*cis*-Sabinol	Wiley
18	15.76	0.82	Verbenyl, ethyl ether	Mainlib
19	16.14	0.48	Myrtenal	Wiley
20	16.26	1.08	Myrtenol	Wiley
21	16.56	2.99	Verbenone	Wiley
22	16.88	1.22	Carveol	Wiley
23	18.19	0.58	3,5-Dimethoxytoluene	Mainlib and Wiley
24	18.63	0.53	Anethole	Wiley
25	18.69	0.60	Bronyl acetate	Mainlib
26	19.39	0.86	5,5-Dimethyl bicyclo [2.1.1] hexane-1-carboxylic acid	Wiley
27	24.36	0.69	2,6-Octadiene-1,8-diol- 2,6-dimethyl	Mainlib and Wiley
28	32.99	0.32	*trans*-Caryophyllene	Wiley
29	33.67	4.05	Neryl nitrile	Mainlib and Wiley
30	33.92	4.61	α-Elemene	Wiley
31	34.29	2.09	Geranyl-alpha-terpinene	Mainlib
32	34.76	0.57	γ-Elemene	Mainlib
33	36.61	0.54	*cis*-α-Bisabolene epoxide	Mainlib
34	37.26	5.24	Methyl arachidonate	Wiley
35	37.34	4.15	Germacrene B	Wiley
36	37.50	6.71	Limonene oxide	Mainlib and Wiley
37	37.63	5.93	15-Oxabicyclo [12.1.0] pentadeca-6,10-diene-7-methanol	Wiley
38	37.95	0.99	Nerolidyl propionate	Mainlib
39	38.56	1.50	9-(3,3-Dimethyloxiran-2-yl)-2,7-dimethylnona-2,6-dien-1-ol	Mainlib and Wiley
40	38.63	0.55	*Z*-5-Methyl-6-heneicosen-11-one	Mainlib
41	38.81	0.45	Nerolidol-epoxy acetate	Wiley
42	39.63	6.52	6-Methyl-5-octen-2-one	Mainlib
43	39.92	0.73	4,8,13-Cyclotetradecatriene-1,3-diol	Mainlib and Wiley
44	40.08	0.95	19-Di-torulosol	Wiley

**Table 2:** Morphometric and statistical investigation of the tested groups.

Item	Group I *	Group II *	Group III *	Group IV *	Group V *	Group VI *
**Area percentage of TNF-α**	14.301 ± 3.590 b,c,d	48.562 ± 5.654 a,c,d,e,f	36.804 ± 2.674 a,b,d,e,f	19.008 ± 2.102 a,b,c,e,f	16.741 ± 1.755 b,c,d	15.141± 2.097 b,c,d
**The color intensity of TNF-α**	10.468 ± 1.746 b,c,d	56.555 ± 4.648 a,c,d,e,f	49.781 ± 2.667 a,b,d,e,f	13.634 ± 2.172 a,b,c,f	12.491 ± 3.164 b,c	10.077 ± 1.548 b,c,d
**Area percentage of COX-2**	14.468 ± 1.744 b,c,d	48.885 ± 4.942 a,c,d,e,f	35.416 ± 2.972 a,b,d,e,f	17.931 ± 2.49 a,b,c	16.611 ± 2.27 b,c	15.618 ± 1.959 b,c
**The color intensity of COX-2**	11.623 ± 1.873 b,c,d	46.024 ± 9.21 a, d,e,f	44.313 ± 8.778 a,d,e,f	14.315 ± 1.568 a,b,c,e,f	12.007 ± 2.172 b,c,d	11.777 ± 1.726 b,c,d
**Area percentage of NF-κb**	10.102 ± 1.359 b,c,d	35.093 ± 2.94 a,c,d,e,f	26.957 ± 4.768 a,b,d,e,f	11.879 ± 0.905 a,b,c,f	11.770 ± 1.137 b,c	9.797 ± 2.204 b,c,d
**The color intensity of NF-κb**	15.016 ± 3.119 b,c,d	57.511 ± 6.423 a,c,d,e,f	43.3 ± 2.281 a,b,d,e,f	20.808 ± 5.3 a,b,c,f	17.722 ± 3.62 b,c	15.764 ± 3.459 b,c,d
**Area percentage of Bcl-2**	48.329 ± 4.509 b,c,d	15.460 ± 3.666 a,d,e,f	21.161 ± 4.128 a,b,d,e,f	42.01 ± 4.137 a,b,c,f	45.047 ± 3.435 b,c	45.933 ± 3.992 b,c,d
**The color intensity of Bcl-2**	46.524 ± 3.213 b,c,d	17.404 ± 4.283 a,d,e,f	25.372 ± 3.233 a,b,d,e,f	42.52 ± 2.448 a,b,c,f	44.091 ± 3.846 b,c	45.465 ± 3.395 b,c,d

* Data are displayed as mean ± SD of the studied group. The letter a denotes significance compared to group I (control); b denotes significance in comparison with group II (diseased); c denotes significance in comparison with group III (diseased, 50 mg/kg FEE); d denotes significance in comparison with group IV (diseased, 100 mg/kg FEE); e denotes significance in comparison with group V (diseased, 150 mg/kg FEE); and f denotes significance in comparison with group VI (150 mg/kg FEE only).

## Data Availability

The data presented in this study are available on request.

## Supplementary Materials

The following are available online at https://www.mdpi.com/article/10.3390/antibiotics10121444/s1, Table S1: Sequences of the utilized primers.
