# Supplementary material for: Antibacterial, Immunomodulatory, and Lung Protective Effects of Boswellia dalzielii Oleoresin Ethanol Extract in Pulmonary Diseases: In Vitro and In Vivo Studies
Source: Antibiotics (Basel). 2021 Nov 25;10(12):1444. doi: 10.3390/antibiotics10121444 (PMC8698344; doi:10.3390/antibiotics10121444)
Supplement: Supplementary file 1 [file antibiotics-10-01444-s001.zip › antibiotics-1432427-SI.pdf]

Antibacterial, Immunomodulatory, and Lung Protective Effects of Boswellia dalzielii Oleoresin Ethanol Extract in Pulmonary Diseases: *In Vitro* and *In Vivo* Studies

Asmaa Saleh [1,2,†], Walaa A. Negm [3,*,†], Engy Elekhnawy [4,*], Thanaa A. El-Masry [5], Walaa S. Elseady [6], Badriyah Alotaibi [1,] , and Suzy A. El-Sherbeni [3]

1 Pharmaceutical Sciences Department, College of Pharmacy, Princess Nourah bint Abdulrahman University, Riyadh 84428, Saudi Arabia;

2 Department of Biochemistry, Faculty of Pharmacy, Al Azhar University, Cairo, Egypt

3 Department of Pharmacognosy, Faculty of Pharmacy, Tanta University, Tanta 31111, Egypt; walaa.negm@pharm.tanta.edu.eg (W.A.N.); suzy.elsherbini@pharm.tanta.edu.eg (S.A.E.-S.)

4 Pharmaceutical Microbiology Department, Faculty of Pharmacy, Tanta University, Tanta 31111, Egypt; engy.ali@pharm.tanta.edu.eg (E.E.)

5 Department of Pharmacology and Toxicology, Faculty of Pharmacy, Tanta University, Tanta 31111, Egypt; thanaa.elmasri@pharm.tanta.edu.eg (T.A.E.-M)

6 Department of Anatomy, Faculty of Medicine, Tanta University, Egypt, Tanta 31111, Egypt; walaa.elssaidi@med.tanta.edu

* Correspondence: walaa.negm@pharm.tanta.edu.eg (W.A.N.); engy.ali@pharm.tanta.edu.eg (E.E.)

† These authors contributed equally to this work.

Table S1. Sequences of the utilized primers.

Gene	Primer sequence*	References
TNF-α	F 5'-ATGAGCACTGAAAGCATGATC-3'<br>R 5'-TCACAGGGCAATGATCCCAAAGTAGACCTGCCC-3'	[49]
IL-6	F 5′-AAAGAGGCACTGGCAGAAAA-3′<br>R 5′-AGCTCTGGCTTGTTCCTCAC-3′	[50]
iNOS	F 5′-TCTTGGTCAAAGCTGTGCTC-3′<br>R 5′-CATTGCCAAACGTACTGGTC-3′	[32]
COX-2	F 5′-TTCAAATGAGATTGTGGGAAAATTGCT-3′<br>R 5′-AGATCATCTCTGCCTGAGTATCTT-3′	[32]
NF-κB	F 5′-GCGGGAGAGGGGATTCCCTGCGGCCCCG-3′<br>R 5′-CGGGGCCGCAGGGAATCCCCTCTCCCGC-3′	[32]
GAPDH	F 5'-ACCACAGTCCATGCCATCAC-3'<br>R 5'-TCCACCACCCTGTTGCTGTA-3'	[49]

* F stands for forward and R stands for reverse.
